# Label retention and stem cell marker expression in the developing and adult prostate identifies basal and luminal epithelial stem cell subpopulations

**DOI:** 10.1186/s13287-017-0544-z

**Published:** 2017-04-26

**Authors:** Jens Adam Ceder, Tilly Wilhelmina Aalders, Jack Antonius Schalken

**Affiliations:** 1 Department of Translational Medicine, Lund University, Skåne University Hospital, Jan Waldenströms gata 35, CRC 91:10, SE20502 Malmö, Sweden; 2 Department of Urology (Route 267), Radboud University Nijmegen Medical Centre, P.O. Box 9101, 6500 HB Nijmegen, The Netherlands

**Keywords:** Adult stem cells, Organogenesis, Development, Prostate, Label retaining

## Abstract

**Background:**

Prostate cancer is the second most frequent cancer among males worldwide, and most patients with metastatic disease eventually develop therapy-resistant disease. Recent research has suggested the existence of cancer stem-like cells, and that such cells are behind the therapy resistance and progression.

**Methods:**

Here, we have taken advantage of the relatively quiescent nature of stem cells to identify the slow-cycling label-retaining stem cell (LRC) populations of the prostate gland. Mice were pulsed with bromodeoxyuridine (BrdU) during prostate organogenesis, and the LRC populations were then identified and characterized in 5-day-old and in 6-month-old adult animals using immunohistochemistry and immunofluorescence.

**Results:**

Quantification of LRCs in the adult mouse prostate showed that epithelial LRCs were significantly more numerous in prostatic ducts (3.7 ± 0.47% SD) when compared to the proximal (1.4 ± 0.83%) and distal epithelium (0.48 ± 0.08%) of the secretory lobes. LRCs were identified in both the basal and epithelial cell layers of the prostate, and LRCs co-expressed several candidate stem cell markers in a developmental and duct/acini-specific manner, including Sca-1, TROP-2, CD133, CD44, c-kit, and the novel prostate progenitor marker cytokeratin-7. Importantly, a significant proportion of LRCs were localized in the luminal cell layer, the majority in ducts and the proximal prostate, that co-expressed high levels of androgen receptor in the adult prostate.

**Conclusions:**

Our results suggest that there are separate basal and luminal stem cell populations in the prostate, and they open up the possibility that androgen receptor-expressing luminal stem-like cells could function as cancer-initiating and relapse-responsible cells in prostate cancer.

**Electronic supplementary material:**

The online version of this article (doi:10.1186/s13287-017-0544-z) contains supplementary material, which is available to authorized users.

## Background

Prostate cancer (PCa) is the second most frequent cancer among males worldwide [14]. Although almost all patients with metastatic PCa initially respond to androgen deprivation therapy (surgical or medical castration), nearly all patients eventually develop progressive castration-resistant disease; death from PCa is invariably due to resistance to contemporary treatment modalities [17].

Prostate carcinogenesis and the mechanisms responsible for castration-resistant tumor growth are incompletely understood. Research from hematopoietic and solid tumors have suggested the existence of stem-like cancer cells, termed cancer stem cells (CSCs), that are believed to be involved in tumor initiation, metastatic spread, therapy resistance, and progression [30]. Much research is now focused on identifying and characterizing this critical cell population(s) in order to develop more effective therapies.

It has been suggested that tumor-initiating cells of prostate adenocarcinoma originate from adult stem cells (SCs) present in the basal epithelial cell layer of the prostate [9, 21, 34, 40]. It is, however, not known whether CSCs are transformed SCs, or differentiated cancer cells that gain self-renewal capacity, or if prostate luminal SCs exists in addition to basal SCs.

The prostate glandular tissue develops from the outgrowing buds of the embryonic urogenital sinus epithelium during prostate organogenesis [32]. It is believed that SCs in the androgen receptor-negative urogenital sinus epithelium initiate budding in response to urogenital sinus mesenchymal growth factors, triggered by the rise of testicular androgens during development [35, 39]. Cells in the basal layer of the mouse prostate have been shown to possess self-renewal capability and can generate all three cell lineages of the prostate epithelium, including basal, secretory luminal, and neuroendocrine cells [26]. The location and identity of SCs in the adult prostate remains controversial, though, and cells with basal, neuroendocrine, and luminal phenotypes all survive castration to different degrees in PCa patients [15, 37], suggesting that stem-like luminal or neuroendocrine cells may also exist and serve as tumor-initiating or relapse-initiating cells in castration-resistant tumors [12, 20, 27, 31].

A distinctly robust method for identifying tissue SCs takes advantage of the relatively quiescent nature of SCs, where cells are pulsed with a label such as the thymidine nucleoside analog bromodeoxyuridine (BrdU) [1, 4, 16, 22]. Whereas rapidly proliferating, transit-amplifying, and maturing cells will dilute the label during a prolonged chase period, slow-cycling SCs will retain the label and they can then be identified as the label-retaining cells (LRCs). Label retention has been used to identify tissue SCs in many various tissues, including intestine, hair follicles, kidney, and the breast [4, 5, 7, 10, 18, 24].

In an attempt to clarify the identity and the location of slow-cycling prostate epithelial tissue SCs, we examined the location of LRCs in the developing and adult mouse prostate in conjunction with cell lineage markers and candidate stem cell antigens. We hypothesized that a critical proliferative window for maximal labeling of epithelial stem cells would be at the time of embryonic prostate bud induction since this is the time period when epithelial SCs in the urogenital sinus should proliferate to initiate prostate budding. We pulsed embryos in utero with BrdU during this phase, and then chased the cells until after birth and adulthood. We found that a significant proportion of epithelial cells in the prostate lobes retained BrdU and that the highest frequency of LRCs was to be found in the prostate ducts. LRCs were identified in both the basal and epithelial cell layers of the prostate, expressing candidate SC markers in a developmental and duct/acini-specific manner, including the novel prostate SC marker cytokeratin-7 (KRT-7). Importantly, luminal LRCs were identified to express androgen receptor (AR) and KRT-7, and to be present in decreasing frequency from ducts to secretory lobes. Current results suggest that there are separate basal and luminal prostate SC populations, and support the idea that stem-like AR-expressing luminal cells could function as cancer-initiating and relapse-initiating cells in prostate cancer.

## Methods

### Mouse label retention model

CD1 mice (*Mus musculus*) were supplied by Harlan Laboratories and bred at the central animal laboratory (CDL) of Radboud University Medical Center, Nijmegen, the Netherlands. The animal study was approved by the Animal Ethics Committee (DEC) at Radboud University. For generation of BrdU-labeled embryos, six pregnant CD1 mice were injected subcutaneously at embryonic days 16.5 (E16.5) and E17.5 with BrdU (50 μg/g) diluted in phosphate-buffered saline (PBS) in the posterior neck, giving rise to 29 BrdU-labeled male offspring that were euthanized 5 days postnatally (P5) or 6 months after birth (adult). Additionally, offspring from nonlabeled pregnant mice were euthanized (E18.5, P0, P5, and adult; three to twelve males per group). Prostates were then dissected out and either freshly snap frozen in OCT (Tissue-Tek, Sakura, the Netherlands) or fixed (1 h/mm tissue) in 4% paraformaldehyde in PBS and embedded in paraffin according to standard procedure.

### Immunohistochemistry and immunofluorescence

Paraffin- and OCT-embedded specimens were cut into 4-μm thick sections, mounted on SuperFrost Plus slides (Menzel Gläser, Braunschweig, Germany), and subsequently deparaffinized and rehydrated (according to standard immunohistochemical protocols), or fixed in either 4% paraformaldehyde or ice-cold methanol for 10 min (cryosections). Paraffin rehydrated specimens were subjected to antigen retrieval (boiling in 10 mM sodium citrate buffer, pH 6.0, for 10 min). For chromogenic immunohistochemistry (IHC), endogenous peroxidase activity was blocked with 0.3% H_2_O_2_ and 0.1% NaN_3_ in PBS (pH 7.4) for 20 min, and avidin/biotin-binding sites were blocked with an avidin/biotin blocking kit according to the manufacturer’s instructions (Vector Laboratories, CA, USA) before each single or sequential (double) staining. For BrdU detection, sections were subjected to acid treatment (10 min incubation in 1 N HCl on ice, followed by 10 min in 2 N HCl at room temperature, and then 30 min in 2 N HCl at 60 °C) followed by incubation in borate buffer (0.1 M, pH 8.5, 12 min), either before or after blocking (5% normal serum in PBS) and incubation with primary antibodies (diluted in PBS with 1% normal serum). IHC detection was performed sequentially with BrdU detection last, whereas immunofluorescence (IF) was performed by co-incubating primary antibodies against BrdU and endogenous antigens after the acid treatment step. However, the epitope of c-kit was sensitive to the low pH of the acid treatment, and slides were therefore blocked and incubated overnight with the primary antibody prior to the acid and borate treatment. Additionally, a postincubation fixation step (4% paraformaldehyde in PBS, 10 min) was performed immediately after incubation with the primary antibody and washing (PBS). Slides were detected with primary or secondary antibodies (Additional file 1: Supplementary material and methods 1) conjugated with either Alexa-594/488 fluorescent dyes, HRP, or with biotin, followed with streptavidin-AP/HRP complexes as necessary, and developed using NBT/BCIP (Roche Applied Science, Mannheim, Germany) and DAB bright (ImmunoLogic, Duiven, the Netherlands) as chromogens according to manufacturer’s instructions and standard immunohistochemical methods. Counterstaining was performed with DAPI (Life Technologies, USA) or hematoxylin (HTX; Vector Laboratories), and cover-slipped with Prolong Gold (Life Technologies, USA) or with Permount (Vector Laboratories) after dehydration. As a negative control, nonlabeled mouse prostates were used for BrdU, and primary antibodies were omitted for endogenous antigens. The kidney, colon, and testes were used as positive controls (Additional file 2: Figure S1). Additionally, preabsorption using immunizing AR peptide (sc-816 P, Santa Cruz Biotechnology, TX, USA) was performed. Slides were evaluated with light and fluorescent microscopy (Olympus AX70, Leica DC 300 F) and scanned with a high-content microscope (Leica DM16000B).

### Quantitative analysis of LRCs and cellular proliferation

Label-retaining and proliferative indexes were calculated from adult prostates double-stained for BrdU/p63 and Ki67/p63, respectively. The mouse prostates were divided into three regions—ductal, proximal, and distal—for the LRC index, and into two regions for the proliferation index—distal epithelium versus ductal-proximal epithelium. Random high-power magnification fields of epithelium from each region from at least three specimens were quantitated. At least 8000 epithelial cells were counted for each index. The fraction of total, basal, and luminal LRCs in each region was calculated by dividing the number of BrdU-positive cells by the total amount of epithelial cells, and number of double BrdU/p63-positive cells by number of p63-positive basal cells, and number of BrdU-positive but p63-negative luminal cells by the number of p63-negative luminal cells in each region. Similar calculations were performed for total, basal, and luminal fractions of proliferating cells in the two regions for Ki67- and p63-stained sections. Statistical comparisons between groups were performed using two-tailed *Z* tests for two population proportions.

## Results

### Proliferation and SC markers are inversely expressed during early prostate development

In male CD1 mice (21 days of gestation), phenotypic prostate glandular development starts with epithelial budding into the mesenchyme at fetal day 17.5. We pulse-chased mice with the synthetic thymidine analog BrdU in order to identify the prostate SC populations. To increase the uptake of the label into the nucleic acid of SCs and to maximize the wash-out of the label in non-SCs, we posited that the label should be given at the time of induction of prostate SC proliferation and epithelial budding, and when few cell divisions have occurred in the developing organ. Additionally, we hypothesized that epithelial SC proliferation may commence prior to phenotypic budding, and hence we chose to start our 2-day label protocol 24 h before morphological budding had occurred, thus labeling animals at E16.5 and at E17.5. The presence of LRCs was then investigated in 5-day-old (P5) prostates, a developmental stage where most of the epithelial branching has occurred, and in adult animals, to investigate if LRCs are long-lived.

**Fig. 1:**
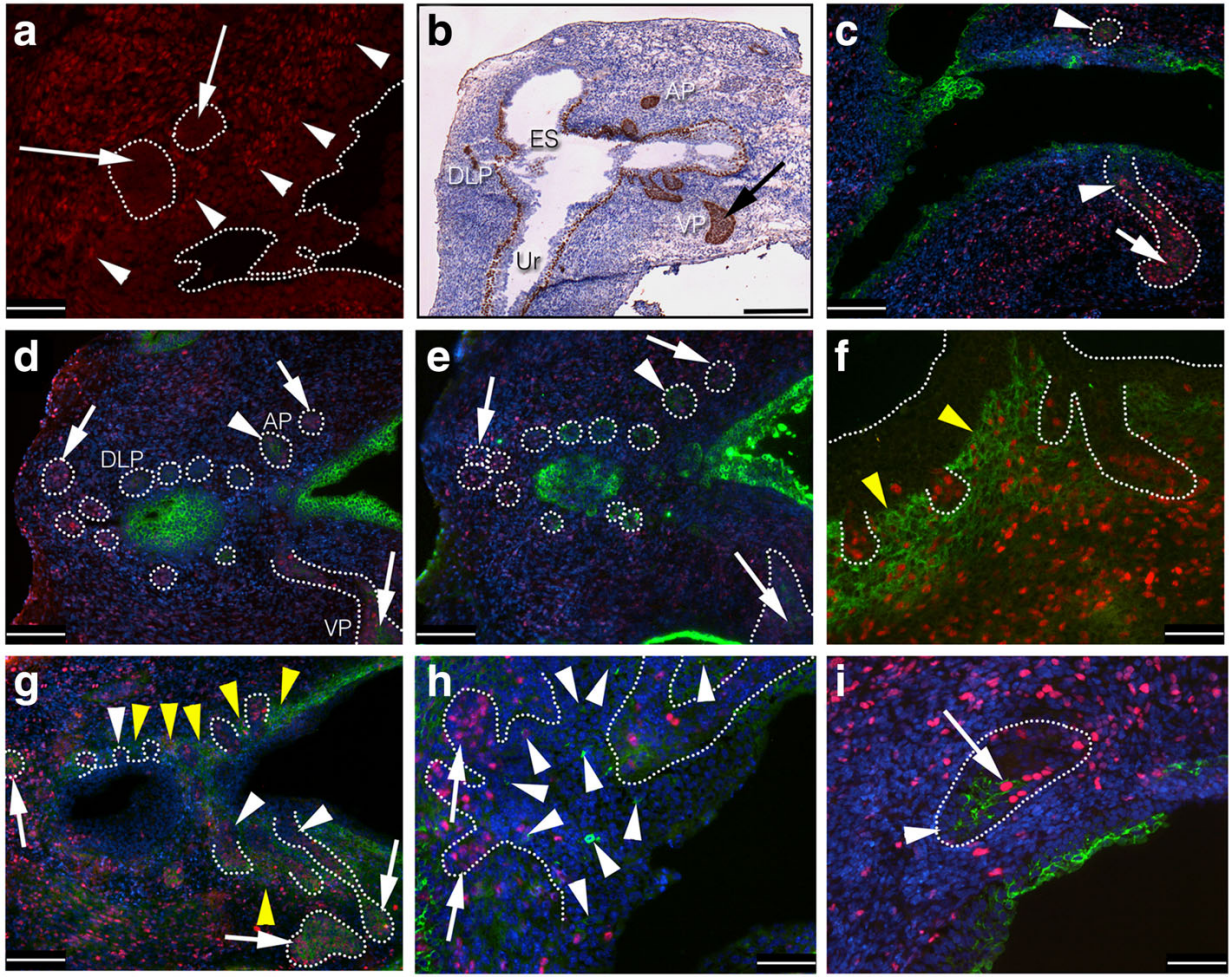
IHC and IF analysis of cell lineage, SC markers, and proliferating cells in early developing mouse prostate. Sagittal sections of urogenital sinus day E18.5 embryo (**a**) and sagittal sections of postnatal day P0 (**b**–**i**) were subjected to antibodies against AR (**a**; *red*), p63 (**b**; DAB), Ki67 (**c**–**i**; *red*), KRT-8 (**c**; *green*), TROP-2 (**d**; *green*), Sca-1 (**e**; *green*), CD133 (**f**; *green*), CD44 (**g**; *green*), c-kit (**h**; *green*), and KRT-7 (**i**; *green*). Note mesenchymal AR expression (*arrowheads*) and absence of epithelial AR (*arrows*) during bud induction (**a**). *Arrow* in (**b**) shows p63 expression in ‘luminally’ located cells of distal epithelium in newborn prostates. Note weak luminally located KRT-8 expression of both proximal (*arrowheads*) and distal epithelium (*arrow*, **c**). Both TROP-2 and Sca-1 showed broader expression in the proximal AP (*arrowhead*, **d, e**) than in the distal AP (*rightmost arrow*, **d, e**) and distal DLP (*leftmost arrow*, **d, e**). Both markers, however, extended into the distal VP (*lower arrow*, **d, e**). CD133 expression was restricted to proximal periurethral mesenchyme (*yellow arrowheads*, **f**). CD44 was not, or only very rarely, detected in duct epithelium (*arrowheads*, **g**), but abundantly expressed in proliferative (**c**–**i**) distal tips (*arrows*, **g**). Note proximal periurethral expression of CD44 in mesenchyme (*yellow arrowheads*, **g**). C-kit was expressed in rare cells, preferentially located in proximal epithelium (*arrowheads*, **h**), the majority negative for Ki67 (*arrows*, **h**). Note a single proliferating KRT-7-positive cell (*arrow*, **i**) at the proximal/intermediate border of the AP (*arrowhead*; proximal epithelium). Counterstaining when used was either HTX (IHC) or DAPI (IF). *Scale bars* = 100 μm (**a**, **f**, **h**–**i**), 200 μm (**c**–**e**, **g**) and 500 μm (**b**); **b** is a composite image of several micrographs. In (**b**), the ventral lobe (*VP*), anterior prostate (*AP*), dorso-lateral prostate (*DLP*), ejaculatory sinus (*ES*), and urethra (*Ur*) are indicated

Since many proteins were sensitive to the harsh treatment of the BrdU detection protocol, we first screened prostates from nonlabeled embryonic and newborn animals for potential SC marker expression during early development in conjunction with the proliferative marker Ki67. The mouse prostate comprises four paired (right-left) lobes (the ventral prostate (VP), anterior prostate (AP), lateral prostate (LP), and dorsal prostate (DP); the DP and LP are often grouped together as the DLP) that are located circumferentially around the urethra, where the excretory ducts fuse with the urethral lumen, or, in the case of the DLP, with the urethral lumen and with the ejaculatory sinus (ES) (the ES drains into the urethra and is formed by the fusion of the terminal ducts of the seminal vesicle and the terminal portion of the vas deferens, the paired ejaculatory ducts). During prostate organogenesis, the principal epithelial ducts arise from these urethral/periurethral structures and, in accordance with previous results, we found that epithelial buds were negative for AR expression in E18.5 prostates, whereas the mesenchyme abundantly expressed AR (Fig. 1a). During later development, the epithelial buds give rise to tributary ducts that branch further and form the arborized glandular secretory lobes. In newborn prostates (P0), basally located cells of ducts expressed the adult basal cell lineage marker p63 (Fig. 1b), and p63 was further expressed by ‘luminally’ located cells of distal epithelium of the lobes, including the VP (Fig. 1b, arrow), likely reflecting an undifferentiated state and the very high proliferative activity (Fig. 1c, arrow) detected in the tip epithelium (Fig. 1c–i). Expression of cytokeratin-8 (KRT-8), a marker of mature luminal cells, was stronger in luminally located cells of both ducts and distal epithelium, however (Fig. 1c, arrowhead). Detection of the proliferative marker Ki67 in P0 prostates indicated that proliferating cells were more abundant in the distal tips in all four lobes, as compared to the terminal portions of the ducts, proximal to the urethra (Fig. 1c–i). A mostly inverse relationship was found for several candidate SC markers, including TROP-2 (Fig. 1d), Sca-1 (Fig. 1e), c-kit (Fig. 1h), and cytokeratin-7 (KRT-7) (Fig. 1i), with stronger SC marker expression proximally than distally, leaving the majority of proximal SC marker-expressing cells negative for the proliferative marker Ki67. However, rare proximal to intermediate located cells positive for the above SC markers did co-stain for Ki67 (Fig. 1i, arrow). In stark contrast, the putative SC protein CD44 was not, or only very rarely, detected in duct epithelium, but was abundantly expressed in distal tips, where a large proportion of cells co-expressed CD44 and Ki67 (Fig. 1g). In addition, the VP expressed TROP-2 and Sca-1 abundantly in distal cells, preferentially luminally located (Fig. 1d and e), whereas the other lobes only harbored rare cells positive for TROP-2, Sca-1, c-kit, and KRT-7 in the distal epithelium. Several SC proteins further showed a dual mesenchymal-epithelial expression pattern, or were restricted entirely to the mesenchyme at this early developmental stage. CD133 was expressed in the ES and periurethral mesenchyme surrounding at least the proximal part of the epithelial buds of all lobes (Fig. 1f, yellow arrowheads). CD44 exhibited the same expression pattern in proximal mesenchyme (Fig. 1g, yellow arrowheads) as CD133, i.e., stronger proximally than distally, and inverse to the epithelial expression pattern of CD44. The mesenchymal expression of c-kit was, on the other hand, stronger in distal mesenchyme as compared to proximal mesenchyme (Fig. 1h, and results not shown), with numerous proliferating cells. Epithelial c-kit expression was rare, and was preferentially located in terminal ducts rather than in distal epithelium, with only exceptional co-staining for Ki67 (Fig. 1h). At this stage, the urothelium of the urethra was largely negative (<1%) for proliferating cells (Fig. 1c–i).

### LRCs and SC markers are enriched in the prostate ducts

**Fig. 2:**
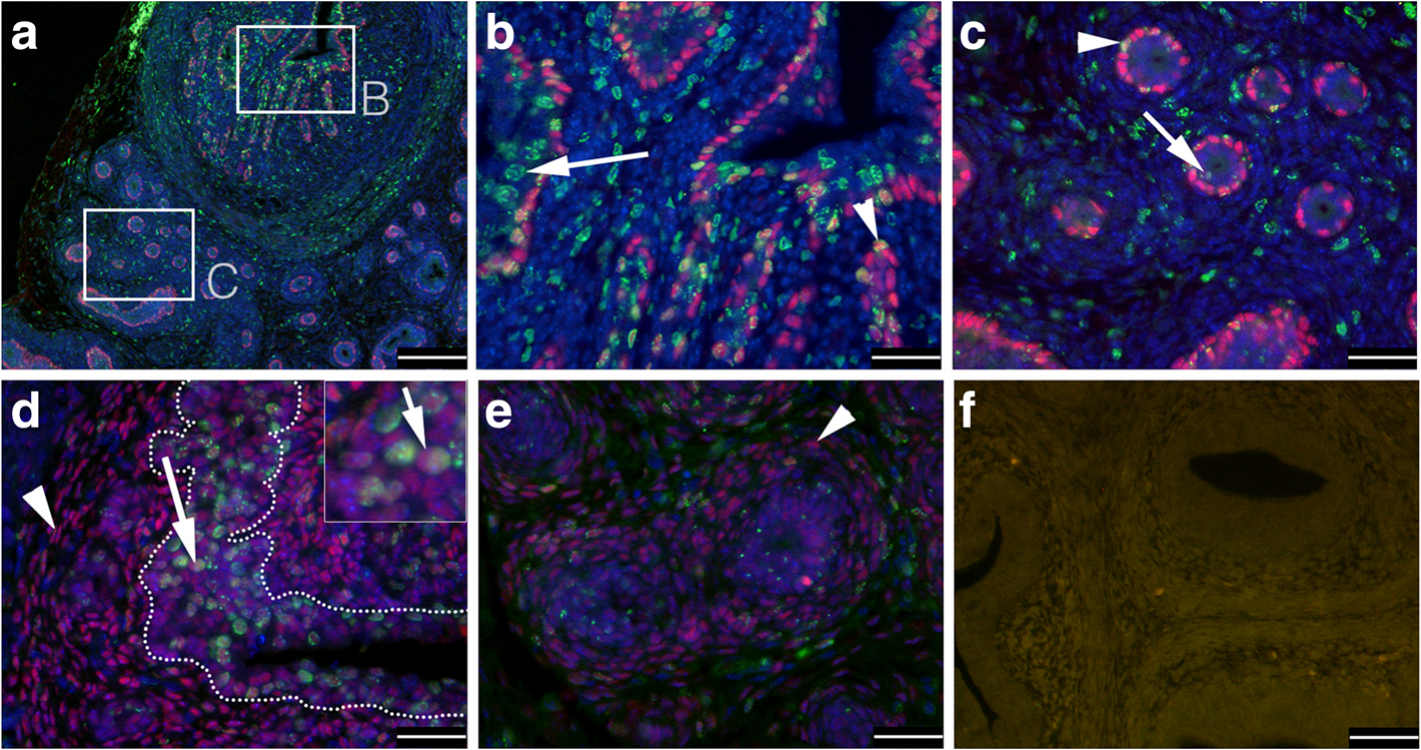
IF analysis of the distribution of LRCs and co-expression of cell lineage markers in transversal sections of postnatal day 5 (P5) mouse prostates. Paraffin sections were subjected to antibodies against BrdU (**a–e**; *green*), p63 (**a–c**), AR (**d, e**), and negative control (**f**). Note that both basally (p63-positive; *arrowheads*) and luminally (p63-negative; *arrows*) located LRCs were more numerous and more strongly labeled in ducts (**a,b**) than when compared to distal epithelium (**a, c**). In P5 prostates, AR was expressed in terminal ducts (**d**), and some of the strongest AR-expressing cells co-expressed BrdU (*arrow*, **d**). However, distal acini (**e**) expressed no or only low levels of AR (*arrowheads* indicate stromal AR expression in **d, e**). DAPI was used as counterstaining in **a–e**. *Scale bars* = 200 μm (**a**), 100 μm (**f**), and 50 μm (**b–e**)

Next we investigated the distribution of LRCs in postnatal day 5 (P5) prostates. The distribution of LRCs in transversal sections of P5 prostates showed a proximal-distal gradient (Fig. 2), with more numerous and more strongly labeled cells in ducts (Fig. 2a and b) than when compared to distal acinar structures of the prostatic lobes (Fig. 2a and c). Whereas many urethral and ductal LRCs would retain the label in the entire nucleus (Fig. 2b), the BrdU content of the most distal LRCs decreased to parts or speckles in the nucleus (Fig. 2e). However, rare LRCs in both the basal and luminal layer of distal acini also retained BrdU in the entire nucleus (Fig. 2c), with basal LRCs being more common than luminal LRCs.

**Fig. 3:**
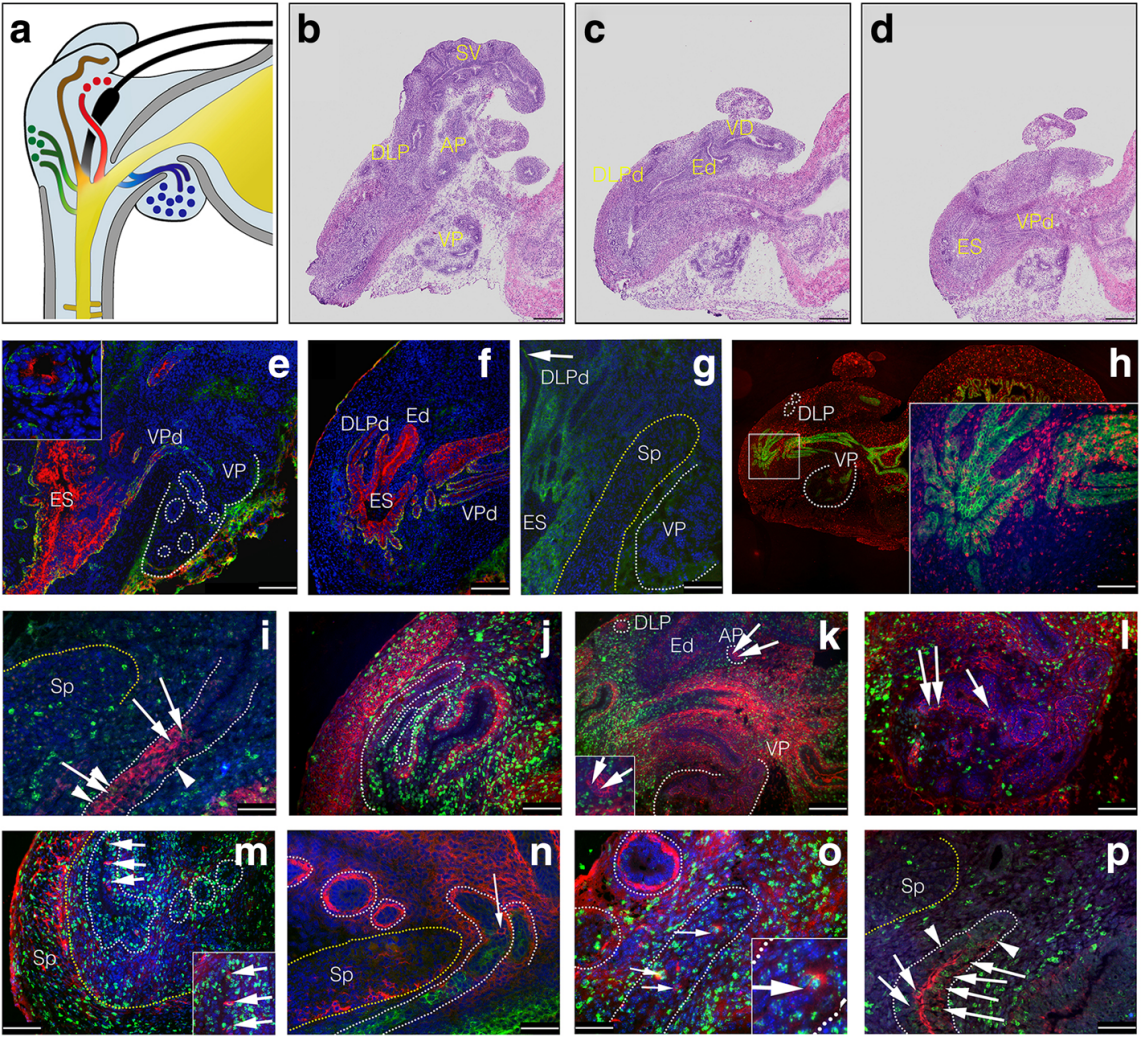
IF analysis of the distribution of LRCs and co-expression of SC markers in sagittal sections of postnatal day 5 (P5) mouse prostates. **a** A cartoon of a sagittal P5 section; bladder and urethra (indicated in *yellow*), stroma (*light blue*), sphincter (*Sp*; *grey*), vas deferens (*VD*) and ejaculatory duct (*Ed*) (both *black*), ejaculatory sinus (*ES*; *orange*), seminal vesicle (*SV*; *brown*), and the ventral prostate (*VP*; *dark blue*), dorso-lateral prostate (*DLP*; *green*), and anterior prostate (*AP*; *red*). **b**–**d** are composite images of serial sagittal HTX stained sections, where ducts draining from a specific lobe are indicated by the addition of a ‘*d*’ to the lobular abbreviation. Frozen sections were subjected to antibodies against Sca-1 (*red*) and CK14 (*green*) (**e**, **f**), CD133 (*green*) (**g**), TROP-2 (*green*) and BrdU (*red*) (**h**), Sca-1 (*red*) and BrdU (*green*) (**i**), CD44 (*red*) and BrdU (*green*) (**j**–**l**), c-kit (*red*) and BrdU (*green*) (**m**, **o**), c-kit (*red*) and Sca-1 (*green*) (**n**), and KRT-7 (*red*) and BrdU (*green*) (**p**). Sca-1 (**e**, **f**, **i**) and TROP-2 (**h**) were highly expressed in basal and luminal cells of ducts draining into the ES and urethra. The mesenchyme surrounding these proximal ducts was positive for CD133 expression (**g**). Sagittal sections clearly demonstrated that LRCs were more numerous and more strongly labeled in terminal draining ducts in or close to where the ES fuses with the urethra (**h**, **j**, **k**), whereas more distal structures contained fewer and less bright LRCs (**h**, **k**). The number and intensity of epithelial LRCs further abruptly decreased at the border of the Sp, where the ducts cross to enter the prostate lobes (**i**, **p**; *arrows* indicate luminal, whereas *arrowheads* indicates a basal location). At this border, many putative SC markers changed their distribution pattern in both epithelium and stroma, including Sca-1 (**i**, **n**), TROP-2 (**h**), CD44 (**k**), c-kit (**n**), and KRT-7 (**p**). Whereas Sca-1 (inset in (**e**) shows rare luminal Sca-1 expression of the VP from a serial section close to (**e**)), c-kit (**n**), and KRT-7 (**p**) expression was rare in distal epithelium of all lobes, we found TROP-2 to be more commonly expressed in the VP when compared to other lobes (**h**). CD133 was upregulated in the distal DLP (*arrow*, **g**) of P5 prostates, and whereas CD44 showed high expression in distal epithelium of the DLP (**k**) and AP (*arrows* and inset in **k**), CD44 was downregulated in distal luminal cells of the VP (**k, l**), but not in the basal cells (*arrows*, **l**). Additionally, VP ducts showed upregulation of CD44 in the basal cell layer (**j**, **k**) in P5 prostates. LRCs strongly reactive for BrdU in the prostate epithelium were found to express the putative SC markers TROP-2 (**h**), Sca-1 (**i**), CD44 (**j**–**l**), c-kit (**m**,**o**), and KRT-7 (**p**). Counterstaining was either HTX (IHC) or DAPI (IF). *Scale bars* = 25 μm (**i**, **o**, **p**), 50 μm (**h**, **j**, **l **–**n**), and 100 μm (**e**–**g**, **k**). **b**–**e** are composite images of several micrographs

In P5 prostates, it was observed that the expression of p63 was downregulated in most luminally located cells, and almost entirely restricted to the basal cell layer of all lobes (Fig. 2c). KRT-8 was also more highly expressed as compared to earlier in development (results not shown), and the AR was upregulated in terminal ducts (Fig. 2d), but not, or only weakly so, in distal acini (Fig. 2e). Interestingly, LRCs in the terminal ducts expressed AR (Fig. 2d). In sagittal sections (Fig. 3), it was clearly demonstrated that LRCs were more numerous and more strongly labeled in terminal draining ducts in, or close to, where the ES fuses with the urethra, whereas more distal structures contained fewer and less bright LRCs (Fig. 3h and k). This pattern was also reflected in the expression pattern of the putative SC markers Sca-1 (Fig. 3e, f, and i), TROP-2 (Fig. 3h), and KRT-7 (Fig. 3p), with strong expression in the main ducts, but with an abrupt downregulation where the ducts cross the apical border of the sphincter urethrae muscle to enter the prostate lobes (Fig. 3i and p). In the prostate lobes, these markers were more rare, and typically strongest in luminal cells (e.g., Fig. 3e). However, in the case of the VP lobes, TROP-2 expression, albeit at lower levels than ductal expression, extended some distance further into the lobes proper (Fig. 3h). CD133 remained strongly expressed in the mesenchyme surrounding the ES and the draining prostate ducts of P5 prostates (Fig. 3g), but was now mildly upregulated in the most distal epithelium of the DLP (Fig. 3g, arrow). Likewise, CD44 was highly expressed in proximal mesenchyme, but weakly (Fig. 3j and k) in mesenchyme distal to the sphincter. The inverse pattern was broadly seen for epithelial CD44 expression, i.e., higher expression in distal epithelium of the AP and DLP (Fig. 3k). However, CD44 was downregulated in the distal luminal cells of the VP, but remained strongly expressed in the basal cell layer of the VP (Fig. 3k and l). Additionally, ducts of the VP now had upregulated CD44 in the basal cell layer (Fig. 3k), but with fewer CD44-positive cells where the ducts fuse with the urethra (Fig. 3j). The stromal expression of c-kit was, on the other hand, stronger distally to the sphincter (Fig. 3n and o) than proximally (Fig. 3m), and was strongly upregulated in the peri-glandular mesenchyme surrounding the epithelial cords of the DLP (Fig. 3n and o). LRCs strongly reactive for BrdU in ductal and proximal epithelium were positive for c-kit expression (Fig. 3m and o). No LRCs positive for the neuroendocrine marker chromogranin A could be identified (Additional file 2: Figure S1).

### Long-lived LRCs are found in both basal and luminal epithelium

**Fig. 4:**
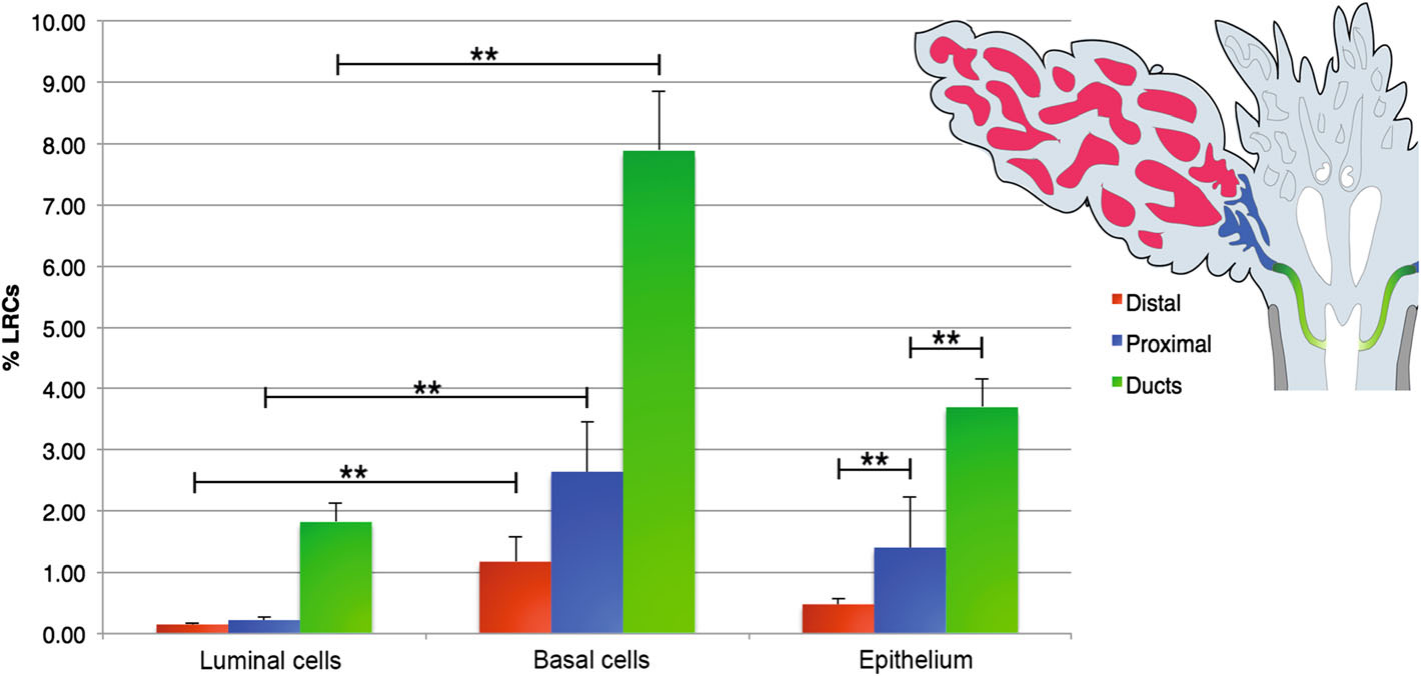
Quantitative analysis of label-retaining cell (*LRC*) distribution in the adult prostate. Quantification of LRCs in the adult mouse prostate showed that epithelial LRCs were significantly more numerous in prostatic ducts (*green*) (3.7 ± 0.47%, mean ± SD) as compared to both proximal (*blue*) and distal (*red*) lobular epithelium, and that proximal epithelium contained significantly more LRCs than distal epithelium (1.40 ± 0.83% vs. 0.48 ± 0.08%). Importantly, both basal and luminal LRCs were detected in the secretory epithelium and ducts of the adult prostate. Basal LRCs in the adult prostate varied significantly between ducts and proximal epithelium (7.89 ± 0.97% vs. 2.64 ± 0.81%, *p* < 0.01), and between proximal and distal secretory epithelium (2.64 ± 0.81 vs. 1.17 ± 0.42%, *p* < 0.01). Likewise, luminal LRCs varied significantly between ducts and proximal/distal epithelium (1.82 ± 0.31% vs. 0.22 ± 0.04%, *p* < 0.01; and 1.82 ± 0.31% vs. 0.15 ± 0.02%, *p* < 0.01), but not significantly between proximal and distal epithelium. Error bars represent standard deviation (SD). **p ≤ 0.01

Next we wanted to investigate whether LRCs are long-lived, and phenotype their adult expression. The distribution of LRCs in the adult prostate was similar to that of the P5 prostates, with more numerous and more strongly labeled LRCs in prostatic ducts as compared to the secretory epithelium. We found that 0.48 ± 0.08% (mean ± SD) of the distal epithelium of the prostate lobes contained long-lived and slow-cycling LRCs, increasing significantly (*p* < 0.01) to 1.40 ± 0.83% in the proximal secretory epithelium of the adult prostate, and that ductal epithelium contained significantly more LRCs than proximal lobes (3.7 ± 0.47% vs. 1.40 ± 0.83%, *p* < 0.01). Importantly, both basal and luminal LRCs were detected in the secretory epithelium and ducts of the adult prostate. Basal LRCs in the adult prostate varied significantly between ducts and proximal epithelium (7.89 ± 0.97% vs. 2.64 ± 0.81%, *p* < 0.01), and between proximal and distal secretory epithelium (2.64 ± 0.81% vs. 1.17 ± 0.42%, *p* < 0.01). Likewise, significantly more luminal LRCs could be found in ducts when compared to secretory lobes (ductal 1.82 ± 0.31% vs. proximal 0.22 ± 0.04, *p* < 0.01; and ductal 1.82 ± 0.31% vs. distal 0.15 ± 0.02%, *p* < 0.01), but not between proximal and distal epithelium (Fig. 4).

**Fig. 5:**
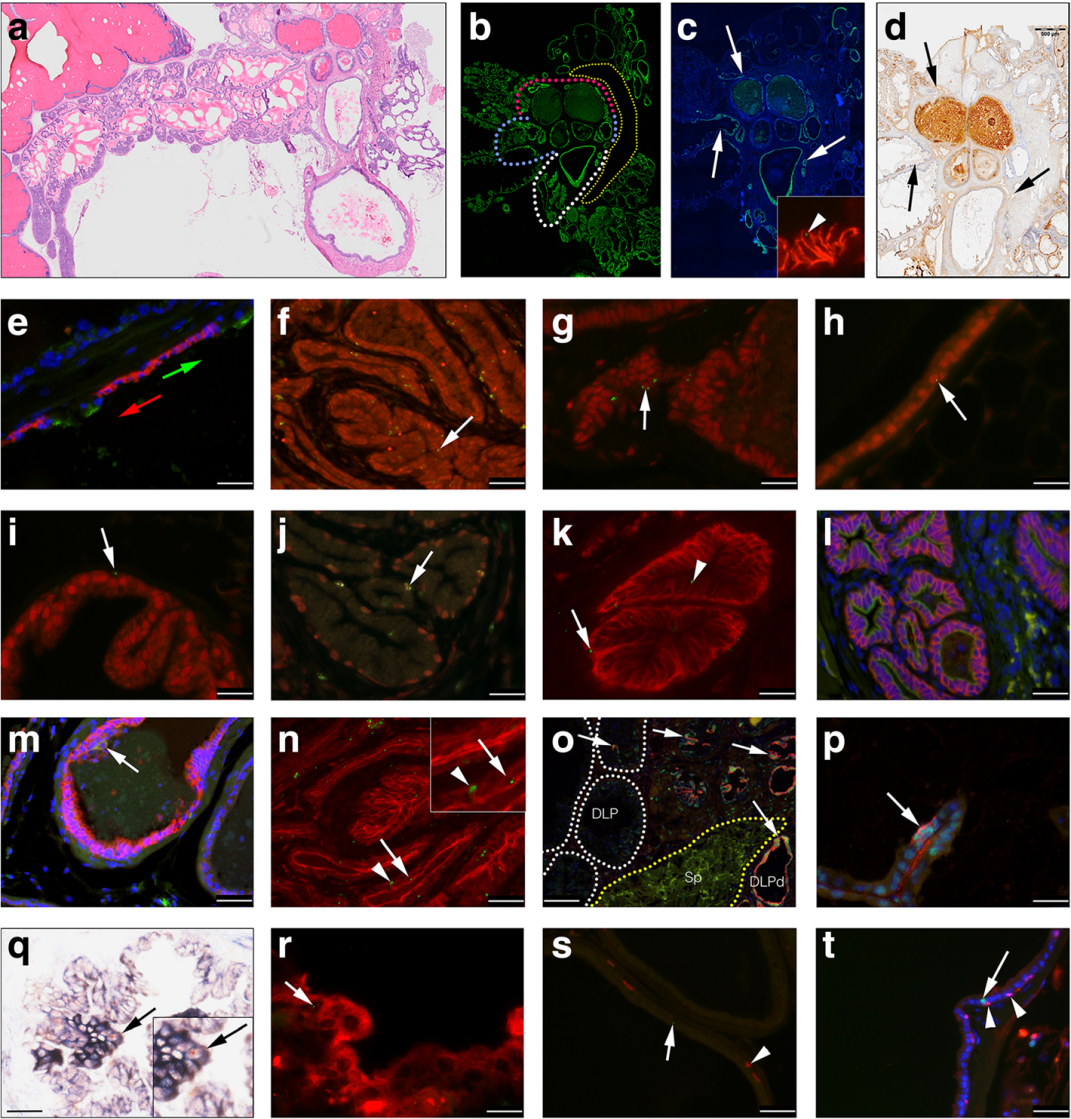
IF and IHC analysis of the distribution of long-lived LRCs and expression of cell lineage and SC markers in the adult mouse prostate. **a** A transversal HTX stained section of an adult prostate; (**b**) shows KRT-8 expression (*green*) in a consecutive section to (**a**), with *dotted lines* marking the border between distal and proximal/ductal epithelium; the *white line* demarks the proximal VP, *blue line* demarks the proximal AP, and the DLP is demarked in *red*. A *yellow dotted line* demarks the sphincter muscle. In (**c**), the abrupt decrease in TROP-2 (*green*) at the proximal-distal border is indicated by *arrows*, and a representational magnification of BrdU (*green*) and TROP-2 (*red*) from the proximal AP is highlighted in the inset (*arrowhead*, **c**). In contrast, CD133 (DAB) was absent from the proximal lobes and ducts (*arrow*, **d**), but strongly expressed by luminal cells of the distal lobes (**d**). CD133 (*green*) and TROP-2 (*red*) expression showed some overlap at the very proximal-distal border of the AP lobes (*green arrow* points distally), but was mutually excluding at the cellular level (**e**). **f**, **g** LRCs (*green*) positive for AR expression (*red*) (*arrows*, **f**–**h**). The *arrow* in (**i**) highlights a basal LRC (*green*) that is negative for AR. Basal LRCs (*green*) expressed p63 (*red*) (*arrow*, **j**), and both basal (*arrow*) and luminal (*arrowhead*) LRCs (*green*) in ducts were found to express CD44 (*red*). **l** KRT-7 (*green*) and TROP-2 (*red*) were co-expressed in prostate ducts. However, KRT-7 (*green*) had a more restricted expression pattern in distal epithelium (**m**) as compared to TROP-2 (*red*). Basal (*arrowhead*) and luminal (*arrow*) LRCs (*green*) co-expressed KRT-7 (*red*) in prostate ducts (**n**). **o** Co-expression of KRT-7 (*red*) and AR (*green*) is seen in the dorso-lateral prostate (*DLP*) (*arrows*); distal DLP epithelium is indicated by *white dotted lines*, and the sphincter muscle (Sp) is demarked by a *yellow dotted line*. Note a rare KRT-7 and AR co-expressing cell in the distal DLP and the high frequency of co-expressing cells in the ducts and proximal DLP (**o**). Rare KRT-7-expressing cells (*red*) in the distal AP further co-expressed high levels of AR (*green*) (*arrow*, **p**). The expression level of c-kit was lower in adult prostates and the harsh BrdU protocol hindered proper IF detection of c-kit; however, using double sequential chromogenic detection, c-kit (NBT/BCIP)-positive LRCs (DAB) could be detected (*arrow*, **q**). **r** A single LRC (*green*) co-expressing Sca-1 is indicated in the distal VP (*arrow*). No LRCs (*green*) (*arrow*, **s**) were found positive for the proliferative marker Ki67 (*red*) (*arrowhead*, **s**). The majority of proliferating (Ki67; *green*) cells were found in the luminal cell layer (*arrow*, **t**) and were negative for p63 (*red*) (*arrowheads*, **t**) expression. Counterstaining when applied was either with HTX (IHC) or DAPI (IF). **a**–**d** are consecutive composite images of several individual photomicrographs. *Scale bar* = 500 μm (**a**–**d**), 25 μm (**i**, **o**, **p**, **r**), 50 μm (**h**, **j**, **l**–**n**, **q**), and 100 μm (**e**–**g**, **k**, **s-t**)

In an effort to better characterize adult basal and luminal LRCs, we co-stained LRCs for cell lineage and SC markers, and investigated SC protein expression in luminal cells (Fig. 5). In adult prostates, KRT-8 was firmly upregulated in virtually all luminal cells regardless of localization (Fig. 5b), whereas candidate SC proteins exhibited a restricted expression pattern broadly similar to postnatal day 5 prostates, with a higher expression in ducts and proximal lobes compared to distal glandular cells (Fig. 5c; TROP-2). A notable exception was CD133, which was strongly upregulated in distal luminal cells of the adult prostate (Fig. 5d). CD133 was, however, not expressed in the LRC-rich ducts and proximal segments of prostate lobes (Fig. 5d, arrows). In fact, CD133 and TROP-2 were mutually excluding at the proximal-distal border of the AP (Fig. 5e). Although TROP-2 did not differentiate between basal and luminal cells, we noted that luminal TROP-2-positive cells showed stronger expression levels in lobes and glands that were heterogeneous in their AR expression (DLP and AP, but not VP). We therefore investigated if adult LRCs expressed AR. Indeed, in ductal (Fig. 5f), proximal (Fig. 5g), and distal epithelium, luminal LRCs were found to express AR, but basal LRCs did not (Fig. 5i). Basal LRCs expressed p63, however, (Fig. 5j) and both basal and luminal LRCs in ducts were found to express CD44, which was highly upregulated in adult ducts and proximal lobes (Fig. 5k), and only rarely expressed in distal epithelium. However, like TROP-2, CD44 was often focal when present, and both markers extended some distance into the distal lobular epithelium of the VP and LP, respectively (Fig. 5c and results not shown). We then turned to KRT-7, which had a similar (Fig. 5l) but more restricted expression pattern compared to TROP-2 (Fig. 5m). KRT-7 was expressed by both luminal cells (strong) and basal cells (weak) of ducts (Fig. 5l), and, similar to TROP-2, KRT-7 expression extended some distance into the VP. However, in the lobes of the DP, LP, and AP, KRT-7 expression was restricted to rare luminal cells. Co-staining BrdU for KRT-7 revealed many basal and luminal LRCs positive for KRT-7 in the prostate ducts (Fig. 5n). However, the harsh BrdU detection protocol hindered the detection of distal KRT-7-positive LRCs, as distal cells generally expressed lower levels of KRT-7 and distal cells seemed more sensitive to the acid treatment. We also noted that ducts stained stronger for the nuclear counterstain DAPI when compared to secretory lobes, possibly reflecting a different chromatin state between nonsecretory progenitor cells and mature epithelium. We then investigated if distal KRT-7 cells were positive for AR expression. Rare distal cells positive for KRT-7 in the DLP (Fig. 5o) and AP typically expressed higher levels of AR. Remarkably, both proximal lobes and ducts of the DLP (Fig. 5o) and the AP showed higher expression of AR in KRT-7-positive cells as compared to KRT-7-negative cells of distal segments (Fig. 5o). We further noted that the number of c-kit-positive cells, like the number of cells positive for other investigated SC markers, increased in absolute numbers in the expanded adult ducts and proximal lobes, and were either found as single cells or small clusters of cells, with rare LRCs expressing c-kit (Fig. 5q, arrow). Similarly, the total number of Sca-1 cells was increased in ducts, and, like TROP-2, Sca-1 expression extended some distance into the distal part of the VP, where rare co-expression of Sca-1 and BrdU could be detected (Fig. 5r). Next we investigated if adult LRCs proliferate, and if proliferative cells are differentially distributed in the adult prostate. However, no LRCs positive for the proliferative marker Ki-67 could be detected (Fig. 5s), and no statistically significant difference was found in the proliferative index between proximal and distal cells (mean 0.46%, range 0.29–0.49%), or between basal and luminal cells (Additional file 3: Table S1). However, since luminal cells are more numerous than basal cells in all prostate lobes (*p* < 0.01), this suggests that luminal cells contribute in a proportionally higher degree than basal cells to cell renewal in the adult mouse prostate (Fig. 5t).

## Discussion

Label retention has previously been used to identify slow-cycling label-retaining stem cells in various organs, including the slow-cycling SCs of the small intestine [4, 5, 7, 10, 18, 24]. We now report the identification of slow-cycling label-retaining cells in both the basal and luminal epithelial cell layers of the mouse prostate, and that they express several SC markers in a lobe and a developmental-specific pattern during organogenesis and maturity, including the novel prostate SC marker KRT-7. Importantly, we found that LRCs were highly expressed in the principal ducts fusing with the urethra and ES, and that this mouse prostate ‘SC niche’ had increased expression of several SC markers, as well as higher expression of the AR compared to secretory epithelium. In addition, both proximal and luminal cells expressing KRT-7 generally expressed AR at higher levels than KRT-7-negative cells in the DLP and AP. Clearly, early (E18.5) prostate epithelial SC are AR negative and do not depend on AR expression during the budding phase, a stage where AR expression is restricted to the mesenchyme. Further, during postnatal development, the AR was upregulated in the nonproliferating terminal ducts, whereas the proliferative distal epithelium had no or only low levels of AR, suggesting that developmental epithelial proliferation does not depend directly on AR activity, in agreement with previous studies [23]. To our knowledge, this is the first time it has been reported that the principal ducts and proximal lobes of the AP and DLP in adult mice express higher levels of AR as compared to distal epithelium. Ductal and proximal cells further stained more strongly for the nuclear stain DAPI as compared to distal epithelium, likely reflecting a different chromatin structure and transcriptional state. We hypothesize that prostate SCs express key stemness epigenetic chromatin-modifying enzymes allowing self-renewal in conjunction with epigenetic modulation of cell-lineage transcription factors, regulating their activity as transcriptional activators or repressors and thus predetermining their cell fate. It is well recognized that the AR has both growth promoting and suppressive activity during development and maturity [25], and that addition of androgens to PCa cell lines induce a bell-shaped proliferative response, where low levels induce proliferation, and higher levels inhibit cell division but stimulate biosynthesis of prostatic fluid components [6, 11]. We have earlier shown that the AR may function directly as a transcriptional repressor by recruiting the repressor protein REST (RE1-silencing transcription factor), and that this complex mediates cell cycle repression, and that REST nuclear displacement in PCa is associated with a poor prognosis [33]. The co-expression of nuclear AR and several SC markers found here further suggests that the AR does not necessarily commit the cell to terminal differentiation, or that the AR downregulates SC genes. Rather, AR may, together with environmental signals and chromatin modulators, mediate transcriptional activation and repression of key genes involved in self-renewal, differentiation, and biosynthesis. Indeed, it has been reported that AR splice variants can upregulate several SC genes in PCa [19]. We propose that AR-mediated repression in luminal progenitors is deregulated early on in prostate carcinogenesis, leading to increased self-renewal and generation of daughter cells. Epigenetic drift may further give rise to clones with different AR transcriptional programs, giving different selective advantages during carcinogenesis and progression, and serve as a basis for heterogeneity in PCa and prostate cancer stem cells [8].

The role of the expression of KRT-7 in prostate SC biology warrants further investigations. Intermediate filament proteins and cytokeratin expression and modulation have been shown to be crucial during development, wound repair, migration, and cell remodeling, as well as in cell division and in neoplasia. Most historical studies have noted that immunoreactivity for KRT-7 in PCa typically occurs in rare individual cells within otherwise nonreactive tumor areas but, due to high cut-off values (5% or higher) in previous studies, most PCas have been reported as negative for KRT-7 [28]. However, Goldstein [13] investigated an unusually large number of PCas and frequency of KRT-7 expression, and reported that KRT-7-positive cases increased with higher Gleason score. In-depth studies on the exact distribution and expression of KRT-7 in primary PCa and in progressing metastatic disease may validate and extend results regarding if KRT7 alone or together with luminal (e.g., AR) or SC surface proteins may serve as a prognostic histopathological or circulating tumor cell marker.

The neuroendocrine cell type is the first cell lineage to differentiate during prostate organogenesis [2], and these cells are thought to be long-lived. By labeling slow-cycling cells, there is a risk that cells that are long-lived and that withdraw from the cell cycle and terminally differentiate during early development will retain the label, and result in ‘over-labeling’ of cells. However, in our experiments, mature neuroendocrine cells expressing chromogranin A were all negative for BrdU, suggesting that this hypothetical limitation does not apply to the current report. Alternatively, neuroendocrine cells form outside the prostate prior to our labeling experiment, and migrate into the prostate during organ formation, as previously suggested [2]. A similar caution for label-retaining assays is that cells that proliferate slowly may not proliferate during the pulse phase, and thus not incorporate the label, and result in ‘under-labeling’, or false negative number of cells—e.g., if adult (injured) organs are pulsed and transit amplifying rather than SCs are recruited for the repair process. Here, we pulsed prostates during organogenesis, and thus hypothetically during maximal tissue-specific SC proliferation. Further, depending on location, tissue-specific SCs may withdraw from the cell cycle at different time points or proliferate more slowly during development. SCs contributing to more proximally located structures in relation to the original SC source/pool may retain more label than SCs that migrate with, and continue to contribute to, structures more distally to the original SC pool during development. This was also seen in our results, explaining the presence of a gradient with stronger and more numerous LRCs more proximally than distally to the urethra, and suggesting that distal LRCs have undergone more cell divisions (‘rapidly proliferating’ SCs) than proximal LRCs. This may have consequences for carcinogenesis, since more distal SCs may have accumulated more mutations. Indeed, in the human prostate, cancer is most frequently developed in the peripheral zone [29].

Our results are consistent with previously reported data in the literature. Tsujimura et al. [36] pulsed and androgen-supplemented castrated (‘injured’) prepubertal animals with BrdU during pubertal development, and reported a proximal-distal gradient of LRCs in the murine prostate. However, they reported that about 50% of epithelial cells (25% each of basal and luminal cells) retained label in the proximal regions of the dorsal prostate, a figure much higher than in the current study. This may be due an ‘over-labeling’ in the previous study, resulting from a too prolonged pulse period in an attempt to effectively label not only transit-amplifying cells but also quiescent SCs in the mature prostate. In the current study, we further report several candidate SC markers expressed by LRCs during development, and that these change dynamically during development. The SC markers differed not only depending on developmental stage, but also on location within the lobes and tissue (proximal-distal). These results suggests that SC proteins may have different roles depending on location and developmental stage, and that caution should be given when using the presence or lack of a given SC marker as identifiers of SC based on conditions other than those investigated. CSCs, for example, are likely to encounter a different microenvironment than normal SCs, and may therefore up- or downregulate proteins reported as tissue-specific SC markers. Our results suggest that the ontogeny of adult prostate SCs and cell lineage-specific cells is a dynamic developmental process, and that not only the basal cell layer but also the luminal epithelium contains slow-cycling LRCs. However, luminal LRCs were generally less intensely labeled than basal LRCs, suggesting that either luminal LRCs are derived from basal LRCs, or that basal and luminal cells have separate progenitors to maintain their lineages, and that luminal LRCs proliferate more frequently during organogenesis. Our results, together with cell lineage tracing experiments, supports the former hypothesis [26], but as suggested by the study by Ousset et al. [26], adult prostate SCs may preferentially be monopotent.

It has previously been reported that castration-resistant cells expressing the homeobox protein Nkx3-1, and that express luminal markers including cytokeratin-18, can survive castration and re-initiate growth [12]. A crucial question is if the human prostate harbors luminal SCs and if such cells are involved in carcinogenesis and relapse of castration-resistant tumors. Notably, in long-term castrated prostates, luminal proliferation becomes more common than basal cell proliferation [38], and the authors suggested that human luminal cells may self-renew.

## Conclusions

Our results suggest that there are separate basal and luminal stem cell populations in the prostate. In castration-resistant PCa, the AR pathway is still active despite androgen deprivation therapy [3]. Our results support the idea that AR-expressing luminal stem-like cells could function as cancer-initiating and relapse-initiating cells in castration-resistant prostate tumors, suggesting that novel strategies targeting this cell population should be considered in the treatment of prostate cancer. Additionally, our results support the notion that neuroendocrine cells are formed prior to prostate organ development.

## Additional files

Additional file 1: Supplementary materials and methods. Antibodies. Describes primary and secondary antibodies, their source, and dilution. (DOCX 14 kb)

Additional file 2: Figure S1. Photomicrographs of positive and negative controls visualized by either immunofluorescence or immunohistochemistry. Figure text descriptions of the photomicrographs found in Figure S1. (ZIP 2981 kb)

Additional file 3: Table S1. Proliferative index in the adult mouse prostate. Describes proliferation indices in the mouse prostate as measured by Ki67 staining. (DOCX 14 kb)

## Abbreviations

AP: Anterior prostate
AR: Androgen receptor
BrdU: Bromodeoxyuridine
CSC: Cancer stem cell
DLP: Dorso-lateral prostate
ES: Ejaculatory sinus
HTX: Hematoxylin
IF: Immunofluorescence
IHC: Immunohistochemistry
LRC: Label-retaining cell
PBS: Phosphate-buffered saline
PCa: Prostate cancer
SC: Stem cell
VP: Ventral prostate
