# Supplementary material for: Label retention and stem cell marker expression in the developing and adult prostate identifies basal and luminal epithelial stem cell subpopulations
Source: Stem Cell Res Ther. 2017 Apr 26;8:95. doi: 10.1186/s13287-017-0544-z (PMC5406885; doi:10.1186/s13287-017-0544-z)
Supplement: Supplementary file 1 — Supplementary materials and methods. Antibodies. Describes primary and secondary antibodies, their source, and dilution. (DOCX 14 kb) [file 13287_2017_544_MOESM1_ESM.docx]

### Additional file 1

### SUPPLEMENTARY MATERIALS AND METHODS 1

**Antibodies**

Primary antibodies against BrdU (clone BMG-6H8, mouse, 1:10, Roche Applied Science, Mannheim, Germany; AB2284, sheep, 1:200, AbCam, Cambridge, UK), TROP-2 (AF1122, goat, 1:30, R&D systems, MN, USA), c-kit (clone ACK2, rat, 1:100, Merck Millipore, MA, USA), CD133 (clone 13A4, rat, 1:50, eBioscience, Aachen, Germany), CD44 (clone IM7, rat, 1:50, BD Pharmingen, San Jose, USA), Sca-1 (AF1226 1:40, goat, R&D systems, Oxon, UK), cytokeratin 7 (clone RCK105, mouse, 1:10, Sanbio BV, Uden, The Netherlands), p63 (clone 4A4, mouse, 1:100, Dako, Glostrup, Denmark), cytokeratin 14 (clone LL002, mouse, 1:80, AbD Serotec, Puchheim, Germany), CgA (clone LK2H10, mouse, 1:100, Merck Millipore, MA, USA; clone DAK-A3, rabbit, 1:1000, Dako, Glostrup, Denmark), AR (sc-816, rabbit, 1:100 and sc-816G, goat, 1:100, Santa Cruz Biotechnology, TX, USA; clone EP670Y, rabbit, 1:100, Abcam, Cambridge, UK), Ki67 (clone SP6, rabbit, 1:200, Thermo Fisher, Fremont, USA; clone MIB-1, mouse, 1:100, Dako, Glostrup, Denmark) and cytokeratin 8 (clone TROMA-I, rat, 1:10, developed by Brulet and Kemler, Institut Pasteur, Paris, France; obtained from the Developmental Studies Hybridoma Bank, developed under the auspices of the NICHD and maintained by the University of Iowa, Department of biology, Iowa City, IA52242) were used. Dilutions are given for immunofluorescence; for immunohistochemistry, antibodies were diluted fivefold to tenfold more depending on chromogen used. In addition, the following conjugated antibodies and detection reagents were used: Biotin conjugated sheep α-BrdU (AB2284, 1:200, AbCam, Cambridge, UK), HRP-conjugated mouse α-BrdU (clone BMG-6H8, 1:10, Roche Applied Science, Mannheim, Germany), HRP- and AP-conjugated streptavidin (S-911 & S-921, 1:4000, Life Technologies, USA), Alexa-488 and Alexa-594 conjugated donkey α-goat/mouse/rat/rabbit/sheep (1:500, Life Technologies, USA), normal donkey serum and biotin conjugated donkey α-goat/mouse/rat/rabbit (1:500) were from Jackson ImmunoResearch Europe, UK.
