# Supplementary material for: Label retention and stem cell marker expression in the developing and adult prostate identifies basal and luminal epithelial stem cell subpopulations
Source: Stem Cell Res Ther. 2017 Apr 26;8:95. doi: 10.1186/s13287-017-0544-z (PMC5406885; doi:10.1186/s13287-017-0544-z)
Supplement: Supplementary file 2 — Photomicrographs of positive and negative controls visualized by either immunofluorescence or immunohistochemistry. Figure text descriptions of the photomicrographs found in Figure S1. (ZIP 2981 kb) [file 13287_2017_544_MOESM2_ESM.zip › SUPPLEMENTARY FIGURE 1 TEXT corrected.docx]

**Figure S1**

### Positive and negative controls of cell lineage and SC markers and analysis of neuroendocrine cell lineage marker expression in LRCs.

### (A-C) are photomicrographs of mouse kidneys, (D-G) testes and epididymidis, (H) colon, and (I) prostate P5 tissue proximal to the urethra. (A-E) and (I) were analyzed with IF, and (F-H) with IHC. The anti-Sca-1 antibody stains parenchymal cells of the kidney (A; red) as previously described. (B) shows CD133 staining (green), whereas (C) shows KRT-7 expression (red) in the kidney. Numerous proliferating cells (Ki67; red) in testes were detected (D), and figure (E) shows co-staining of TROP-2 (green) and AR (red). In (F) strong c-kit reactivity is indicated by brown DAB, and (G) is negative control. Colon tissue was additionally used as a positive control to detect c-kit positive mast cells (brown) in the stroma. Neuroendocrine cells (arrowheads in I) detected with anti-chromogranin A antibodies (I; red) were negative for BrdU (green), indicating that neuroendocrine cells may originate from cells that migrate into the prostate during organogenesis as previously suggested (Aumuller et al., 2001). Counterstaining when applied was either with HTX (IHC) or DAPI (IF). Scale bar is 200 µm (A-C, H), 100 µm (E) 50 µm (D, F-G, I).
