# Supplementary figures and images for: Label retention and stem cell marker expression in the developing and adult prostate identifies basal and luminal epithelial stem cell subpopulations
Source: Stem Cell Res Ther. 2017 Apr 26;8:95. doi: 10.1186/s13287-017-0544-z (PMC5406885; doi:10.1186/s13287-017-0544-z)

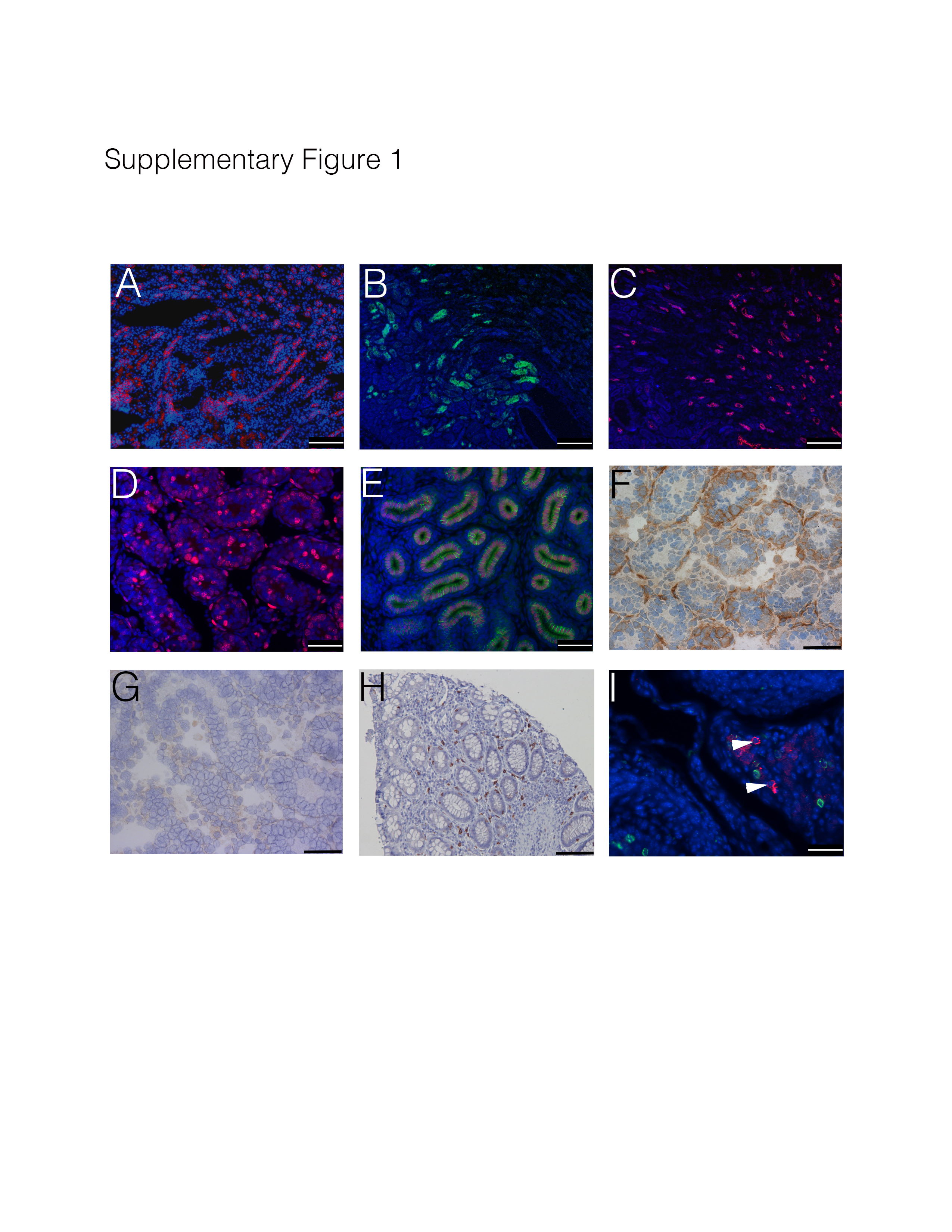

Supplement: Supplementary file 2 — Photomicrographs of positive and negative controls visualized by either immunofluorescence or immunohistochemistry. Figure text descriptions of the photomicrographs found in Figure S1. (ZIP 2981 kb) [file 13287_2017_544_MOESM2_ESM.zip › SUPPLEMENTARY FIGURE 1.jpg]
