# Supplementary material for: Label retention and stem cell marker expression in the developing and adult prostate identifies basal and luminal epithelial stem cell subpopulations
Source: Stem Cell Res Ther. 2017 Apr 26;8:95. doi: 10.1186/s13287-017-0544-z (PMC5406885; doi:10.1186/s13287-017-0544-z)
Supplement: Supplementary file 3 — Proliferative index in the adult mouse prostate. Describes proliferation indices in the mouse prostate as measured by Ki67 staining. (DOCX 14 kb) [file 13287_2017_544_MOESM3_ESM.docx]

### TABLE S1

### Proliferative index in the adult mouse prostate

|  | Epithelium | Basal | Luminal |
| --- | --- | --- | --- |
| Mean | 0.46% (38/8214) | 0.36% (3/820) | 0.47% (35/7394) |
| Proximal | 0.46% (20/4365) | 0.42% (2/476) | 0.46% (18/3889) |
| Distal | 0.47% (18/3849) | 0.29% (1/344) | 0.49% (17/3505) |
|  |  |  |  |

### The mouse prostate contained 0.46% proliferating epithelial cells on average, as measured by Ki67 staining. No statistical significant difference was found in the proliferative index between proximal and distal cells, or between basal and luminal cells.
